# The Implications of Vitamin D Status During Pregnancy on Mother and her Developing Child

**DOI:** 10.3389/fendo.2018.00500

**Published:** 2018-08-31

**Authors:** Carol L. Wagner, Bruce W. Hollis

**Affiliations:** Neonatology, Shawn Jenkins Children's Hospital, Medical University of South Carolina, Charleston, SC, United States

**Keywords:** vitamin D, cholecalciferol, pregnancy, fetal development, health effects, immune mediator, genetic effects, developmental origins of later disease

## Abstract

Pregnancy is a time of tremendous growth and physiological changes for mother and her developing fetus with lifelong implications for the child. The concert of actions that must occur so mother does not reject the foreign tissue of the fetus is substantial. There must be exquisite balance between maternal tolerance to these foreign proteins of paternal origin but also immune surveillance and function such that the mother is not immunocompromised. When this process goes awry, the mother may experience such pregnancy complications as preeclampsia and infections. Vitamin D deficiency affects these processes. Controversy continues with regard to the optimal daily intake of vitamin D, when sunlight exposure should be taken into account, and how to define sufficiency during such vulnerable and critical periods of development. The importance of vitamin D supplementation during pregnancy in preventing some of the health risks to the mother and fetus appears linked to achieving 25(OH)D concentrations >40 ng/mL, the beginning point of the plateau where conversion of the vitamin D metabolite 25(OH)D, the pre-hormone, to 1,25(OH)_2_D, the active hormone, is optimized. Throughout pregnancy, the delivery of adequate vitamin D substrate—through sunlight or supplement—is required to protect both mother and fetus, and when in sufficient supply, favorably impacts the epigenome of the fetus, and in turn, long term health. There is a growing need for future research endeavors to focus not only on critical period(s) from pre-conception through pregnancy, but throughout life to prevent certain epigenetic changes that adversely affect health. There is urgency based on emerging research to correct deficiency and maintain optimal vitamin D status. The impact of vitamin D and its metabolites on genetic signaling during pregnancy in both mother and fetus is an area of great activity and still in its early stages. While vitamin D repletion during pregnancy minimizes the risk of certain adverse outcomes (e.g., preterm birth, asthma, preeclampsia, and gestational diabetes), the mechanisms of how these processes occur are not fully understood. As we intensify our research efforts in these areas. it is only a matter of time that such mechanisms will be defined.

## Introduction

Now three decades later, the established doctrine of the “Barker Hypothesis,” first described by David Barker in 1986, noting a connection between neonatal growth restriction and small for gestational age status with the risk of heart disease later in adult life (1) has become mainstream thinking, but when first proposed was a startling revelation. The theory that diseases that manifest in adulthood actually began during the perinatal period and that the very nutrients and environmental milieu—good and bad—affects our ability to prevent or even fight disease is a daunting idea. It is essential that we understand how early-life exposures can affect later health. One example often given to highlight the effect of perinatal factors is perinatal thyroid function, which is absolutely essential for early-life brain development and maturation. In this example, iodine deficiency and its consequences certainly qualify as an instance of the Barker hypothesis applied. A critical feature of diseases that support the Barker hypothesis is nutritional irreversibility; beyond specific critical points in the developmental cascade, despite full nutrient availability and repletion, the earlier nutritional alteration or deficiency's effect cannot be mitigated.

Perhaps a more publicized nutrient deficiency during the perinatal period is folate deficiency resulting in spina bifida, a neural tube defect that can have catastrophic and certainly life-long sequelae (2). In the 1960's, Hibbard and Smithells established a registry of infants with malformations born in Liverpool, England (3), which included infants with the spina bifida. Through careful study of the infant families, these investigators were able to establish a link between spina bifida and a nutrient-poor diet. Smithells et al went on to define the lack of folic acid, specifically, in the early perinatal period as a contributing factor in the development of spinal bifida (4), a truly remarkable association. The provision of folate beyond the neural tube formation period during gestation does not correct the defect; for those who are folate deficient and with certain genetic risk factors, the provision of adequate folate must occur during the critical period of neural tube formation. As will be discussed later, it appears that with vitamin D deficiency is similar to folate deficiency in that there is a critical period when vitamin D deficiency is more deleterious—for example, placentation can be altered, leading to adverse pregnancy outcomes.

In the past, only the phenotypic result could be observed as a consequence of any given nutritional deficiency, including thyroxine and folate deficiencies. Other nutrient deficiencies, which may impact the long-term health status of the fetus and child, are not so easily discernible by phenotypic changes along. Vitamin D deficiency, long known to be linked to bone mineralization and the development of rickets in its extreme form, was not known until recently to affect not only bone metabolism, but also immune function. Our understanding of nutrient interactions with genes is just beginning to be deciphered. Presently, we have the capabilities of not only observing the phenotype but also the genetic changes contributing to any given phenotype. The focus of this review is to shed light on the potential impact of dietary vitamin D during the prenatal and perinatal periods as a contributor to maternal/infant afflictions, and thus its role in instituting examples of the Barker hypothesis at both the genetic and phenotypic levels. These afflictions include but are not limited to autoimmune disorders, complications of pregnancy, immune function and respiratory disease. We will not be discussing vitamin D metabolism in this text as that information is readily available elsewhere (5, 6).

## What is a “normal” circulating 25(OH)D concentration in humans?

Because the parent compound vitamin D or cholecalciferol has a brief half-life of 12–24 h, it is its metabolite 25-hydroxy-vitamin D or 25(OH)D with its 2–3 weeks-half-life that is used as the indicator of vitamin D status, defining a “normal” circulating concentration of 25(OH)D in humans remains controversial. For instance, while the most recent Institute of Medicine (IOM) concludes that a circulating concentration of 20 ng/mL is adequate to meet human physiological requirements (7), the Endocrine Society suggests that a 25(OH)D concentration of at least 30 ng/mL is linked with better health outcomes (8). The difference is that the IOM recommendation was based exclusively on skeletal integrity data and did not include extraskeletal and immune function vitamin D data (7), whereas the Endocrine Society used both skeletal studies and other studies surrounding vitamin D's immune effects, and included studies that included observational and clinical trials of subjects with various cancers, immune dysfunction, and pregnancy complications, and their associations with vitamin D deficiency (8).

Given the fact that vitamin D is the only preprohormone in the body that is made following sunlight (UV-B) exposure to the skin/epidermis with little contribution from Western and vegetarian diets, defining what is “normal” should include those who regularly have exposure to sunlight. Thus, to define “normal” circulating 25(OH)D status in humans in a meaningful manner, vitamin D sufficiency should be based on 25(OH)D concentrations in “healthy subjects” who are, in fact, exposed to the sun: sunbathers, fieldworkers, and indigenous people living and functioning in environments to which they are native. To reach a circulating concentration of 150 nmol/L (60 ng/mL), which is the concentration of individuals with full access to sunlight living in a sunrich environment achieve, and is the concentration achieved by the sun-exposed lifeguards (5), a dietary intake of 4,000–6,000 IU/d in adults, including the pregnant woman is required (6). Just to be clear, a daily intake of 10,000 vitamin D IU/day in an adult is deemed to be safe (8).

The current RDA of 400–600 IU vitamin D/day unless augmented with sunlight exposure does not achieve this in most adults (9). In fact, the 400 IU/d dose that is recommended by the IOM for older children and adults also is recommended for breastfeeding neonates a few days after birth (7, 10). When taking a dietary supplement of vitamin D containing 400 IU, on a per kilogram basis, the reference newborn infant weighing 3 kg receives ~133 IU/kg and the reference 60 kg pregnant woman receives 6.7 IU/kg. Based on pharmacokinetics, unless the pregnant woman has access to sunlight exposure, her circulating 25(OH)D concentration, which is the gage of her vitamin D status, will be around 37.5–62.5 nmol/L (15–25 ng/mL) whereas the newborn infant receiving 400 IU/day will have achieved circulating 25(OH)D concentrations around 100 nmol/L (40 ng/mL) (11, 12). Because of differences in various factors such as vitamin D binding protein genotype, body mass index, latitude, sunlight exposure, and season, the amount of vitamin D taken orally will result in a wide variety of circulating 25(OH) concentrations among individuals (6). Thus, at this juncture, the only way to know for certain what an individual's vitamin D status is would be to measure that individual's total circulating 25(OH)D concentration.

## Relevant data surrounding vitamin D deficiency during pregnancy: animal models and human studies

### Placental function

Observational studies have linked vitamin D deficiency with preeclampsia in humans (13). Preeclampsia is a condition that complicates up to 10% of pregnancies, 3% with severe, early-onset with potential life-threatening consequences; and is characterized by abnormal placentation and vasculitis in the mother that leads to hypertension, proteinuria and often abnormal liver function affecting growth and development often necessitating early delivery of the fetus, and remains the leading cause of premature delivery (14). The condition can progress to eclampsia or maternal seizures, associated with significant morbidity and mortality. To date, there is no preventive or treatment measure for this condition other than delivery of the fetus. While the etiology of this serious pregnancy affliction remains unknown and without definitive treatment other than delivery of the fetus, there are studies that suggest there is a derangement in placentation and placental function early-on in pregnancy that manifests weeks later. To support this premise are animal studies and observational studies in humans.

Liu et al. (15) using a pregnant mouse preeclampsia model, studied the effect of low vitamin D status on the risk of preeclampsia. Female BL6 mice raised on vitamin D-sufficient or deficient diets were mated with vitamin D-sufficient BL6 males. The resulting pregnant mice were either allowed to deliver and monitored for blood pressure (BP) or euthanized prior to delivery for analysis of serum, placental/kidney tissues, and fetuses. Vitamin D-deficient pregnant mice exhibited both elevated systolic and arterial pressure that continued through pregnancy until 7 days postpartum, returning to baseline at 14 days postpartum. Analysis of maternal kidney samples showed an association between increased renin and the angiotensin II receptor mRNA expression and vitamin D deficiency. Histological analysis of deficient placentas showed decreased vascular diameter within the labyrinth region. Re-supplementation of vitamin D post-conception partially reversed the effects of vitamin D deficiency. Overall, these data provide evidence that low vitamin D status may predispose pregnant women to dysregulated placental development and elevated blood pressure.

The findings of Liu et al. (15) were supported by a recent study by our group involving healthy pregnant women enrolled in a vitamin D supplementation trial. We analyzed placental tissue from 43 women who had participated in a vitamin D supplementation trial. Women had been randomized to 400 IU or 4,400 IU of vitamin D_3_ (cholecalciferol, the parent compound) per day during pregnancy (16). Within 1 h of delivery, placental mRNA was isolated, and analyzed by quantitative PCR for mRNA expression associated with both angiogenesis and vitamin D metabolism. Based on our earlier work where the conversion of 25(OH)D to 1,25(OH)2D was optimized at 25(OH)D concentrations of at least 100 nmol/L (40 ng/mL), the mRNA expression was analyzed on the basis of those women who had achieved this threshold within 1 month of delivery vs. those who had not (11). Soluble FMS-like tyrosine kinase 1 (sFlt-1) and vascular endothelial growth factor (VEGF) gene expression were significantly downregulated in women with circulating 25(OH)D ≥100 nmol/L compared to those with a concentration <100 nmol/L (11). This novel finding suggests that early vitamin D status plays a role in placentation and that this is a critical nutrient threshold concentration. It appears that the impact of maternal vitamin D_3_ supplementation on gene transcription in the placenta, and placentation itself, may be through the balance of such antiangiogenic factors (17, 18).

Consistent with the premise that derangement early-on in placentation and placental growth and function is the finding of incomplete human extra villous trophoblast invasion of the decidua and maternal spiral arteries in pre-eclamptic placentas (19). It is hypothesized that suboptimal vitamin D action on placental tissues leads to extra-villous invasion and altered placentation (20). Chan et al. (20) demonstrated that vitamin D significantly increased extra-villous trophoblast invasion. This was accomplished by increased promatrix metalloproteinases (MMP's), supporting the role of vitamin D in extra-villous trophoblast invasion and preeclampsia. Further studies by this research group have shown that vitamin D is an important component during pregnancy of anti-inflammatory immune responses in the placenta (21). Finally, consistent with the work of Schulz et al. (16), Ma et al. showed that vitamin D supplementation prevents placental ischemia-induced endothelial dysfunction through downregulation of placental soluble FMS-like tyrosine kinase-1, which has been implicated in the pre-eclamptic state (22).

### Neurodevelopment

Vitamin D is a potent neurosteroid which mediates numerous actions in the brain. Localization studies have shown the vitamin D activating enzyme CYP27B1 and catabolic enzyme CYP24A1 in neural cells of the cerebral cortex and cerebellar Purkinje cells, suggesting that the brain is capable of vitamin D metabolism at the local level (23). Eyles et al. (24), in their landmark studies, demonstrated that rats born to vitamin D-deficient mothers had profound alterations in the brain at birth. The cortex was longer but not wider, the lateral ventricles were enlarged, the cortex was proportionally thinner and there was more cell proliferation throughout the brain. There also were reductions in brain content of nerve growth factor and glial cell line-derived neurotrophic factor and reduced expression of p75^NTR^, the low-affinity neurotrophic receptor (24).

Further studies by the Eyles group (25) demonstrated vitamin D-deficiency *in utero* resulted in embryos and pups having significantly less apoptotic cells and more mitotic cells. Hawes et al went on to show more recently that targeted gene arrays specific for apoptosis and cell cycle genes were associated with specific transcriptomic deregulation in the vitamin D-deficient group (26). In this animal model, the investigators also showed an association between vitamin D deficiency during pregnancy and a reduction in fetal crown-rump length, head size, and lateral ventricle volume (26). Brain-derived neurotrophic factor, transforming growth factor-β_1_, and forkhead box protein gene expression also were altered. Further, there was a reduction in Foxp2 immunoreactive cells in the developing cortex associated with vitamin D deficiency. In the substantia nigra, both brain-specific tyrosine hydroxylase gene expression and localization were reduced (26). Taken together, these changes have significant implications for structural and functional alterations in neurodevelopment.

Through various animal models, certain associations have been noted that link vitamin D status with brain development. Vuillermot et al. (27), utilizing a murine model of autism, demonstrated that vitamin D treatment during pregnancy attenuated and/or prevented neurodevelopment disorders following maternal inflammation during pregnancy. The study further demonstrated that prenatal administration of 1,25(OH)_2_D, the active vitamin D hormone, abolished all behavioral defects in polyriboinosinic-polyribocytidylic acid (poly[I:C])-treated juvenile mice with autism (27). Other findings in rodent models have linked maternal vitamin D-deficiency during pregnancy with later spatial learning deficits in the offspring (28) and fertility dysfunction in female mice offspring thought to be due to deleterious effects on the neuroendocrine axis (29).

Extending the findings from animal models to the human realm, Dr. John Cannell in 2008 first proposed the association between vitamin D and Autism Spectrum Disorders (ASD) (30). Included in ASD are autistic disorder, Asperger's Syndrome, Rett's Syndrome, Childhood Disintegrative Disorder, and Pervasive Development Disorders. Epidemiological studies as well as animal models have suggested a potential role for vitamin D deficiency in the development of ASD (27, 31).

### Lung maturation and function

The link between vitamin D deficiency and abnormal development is not limited to the brain. It is known that vitamin D deficiency has risen in the past few decades around the globe (32) and with this rise in deficiency is the concomitant rise in autoimmunity and asthma (33, 34). Vitamin D is known to affect certain genes in the developing lung that are upregulated; and these same genes (for example, matrix mellallopeptidase 9; NF-k light polypeptide gene enhancer in B cells inhibitor; epidermal growth factor receptor; E1A binding protein p300), are linked to the later development of asthma (35).

Zosky, et al, using a relevant BALB/c mouse model of vitamin D deficiency, studied somatic growth, lung function and lung structure at 2 weeks of age (36). It was determined that volume dependence of lung mechanics was altered by vitamin D deficiency, suggesting altered tissue structure. In this model, the primary histological difference between vitamin D-deficient and replete groups manifested in differences in lung volume, supporting the link between vitamin D deficiency and lung development. The same group subsequently published another that described the effects of *in utero* vitamin D deficiency on airway smooth muscle mass and function (35). Using a mouse model, this team showed that there was differential expression of certain gene pathways involved in embryonic organ development, pattern formation, branching morphogenesis, wingless/Int, and inflammation in vitamin D deficient mice, and included genes upregulated in individuals with asthma (e.g., matrix metallo-peptidase 9, NF-κ light polypeptide gene enhancer in B cells inhibitor, an epidermal growth factor receptor and E1A binding protein p300) (35). Vitamin D deficiency in this model also was associated with increased airway smooth muscle mass (ASM) and baseline airway resistance as well as altered lung function (36). Collectively, these data suggest that vitamin D deficiency states during pregnancy are associated with alteration in lung structure and function and increased inflammation, contributing to asthma, which manifests well after birth, and further suggests epigenetic mechanisms of action of vitamin D.

One of the most interesting animal studies comes from Yurt, et al., who investigated the effect of vitamin D supplementation on lung morphology (37). Specifically, using an *in vivo* rat model, the investigators determined the effects of perinatal vitamin D deficiency on overall pulmonary function and tracheal contraction as a functional marker of airway contractility. One month before pregnancy, rat dams were randomized to either a no cholecalciferol-added or a 250, 500, or 1,000 IU/kg cholecalciferol-added diet, which was continued throughout pregnancy and lactation. At postnatal day 21, offspring plasma 25(OH)D concentrations and pulmonary function (as measured by whole body plethysmography and tracheal contraction response to acetylcholine) were determined. In a dose-dependent manner, compared with the 250 and 500 IU/kg vitamin D-supplemented groups, the no cholecalciferol-supplemented group, following a methacholine challenge, showed a significant increase in airway resistance. What was particularly interesting was that the vitamin D deficiency-mediated increase in tracheal contractility was only blocked by supplementation with the higher maternal dose of 500 IU/kg cholecalciferol. Therefore, in addition to altering alveolar epithelial-mesenchymal signaling, perinatal vitamin D deficiency was associated with altered airway contractility. The findings provide some insight into asthma pathogenesis and the mechanistic effects of vitamin D in perinatally vitamin D-deficient offspring, and further suggest that vitamin D could play a role in the prevention of childhood asthma through perinatal vitamin D supplementation, as has been suggested by Litonjua et al and others (38–40).

Other *in vivo* studies support the role of vitamin D in pulmonary function. Taylor, et al, showed promising and provocative results utilizing a neonatal rat model (41). The investigators randomized one-day-old rat pups to one of three different doses of the active hormone, 1,25(OH)D_2_ and its physiologic precursor 25(OH)D, or the diluent, via nebulization daily for 14 days. Compared to controls, nebulized vitamin D-treated group had enhanced lung maturation as evidenced by the increased expression of markers of alveolar epithelial (SP-B, leptin receptor), mesenchymal (PPARγ, C/EBPα), and endothelial (VEGF, FLK-1) differentiation, surfactant phospholipid synthesis, and lung morphology without any significant increases in serum 25(OH)D or in serum calcium concentration.

## Vitamin D deficiency during pregnancy: human studies

### Observational studies

For decades, vitamin D was thought to be important to the pregnant woman and her developing fetus only for the maintenance of calcium homeostasis and skeletal integrity. Research during those decades was directed at answering the question of what was necessary to prevent hypocalcemia and prevent bone loss or underdevelopment in the mother and her newborn. Whether it was serendipity or the realization among certain physicians and scientists that vitamin D was involved in other physiological processes, studies began to emerge that linked vitamin D deficiency with long-latency diseases and immune dysregulation.

This new avenue of research extended to the question of whether or not the entity of preeclampsia, which is the leading cause of preterm birth in many parts of the world, was linked in any way to vitamin D deficiency. Reports began to emerge that found an association between the dietary vitamin D_3_ intake in pregnant women and preeclampsia (42). Looking to historical studies, it is interesting that now more than half century ago, Olsen and Secher (42), in their studies of pregnant women given halibut liver oil, a rich source of vitamin D_3_, found that this supplementation was associated with decreases in preterm birth and preeclampsia, which the authors attributed to marine n-3 fatty acids, with no mention of vitamin D and its potential effect (42). This perspective in the early 1940's was based on the then unknown effects of vitamin D on various systems besides calcium and bone.

More recent studies have delineated vitamin D's extraskeletal and immunological effects, which has brought the question of vitamin D's importance during pregnancy into a new light (38, 43–57). Early observational studies suggested a consistent relationship between maternal circulating concentrations of 25(OH)D and preeclampsia (13, 51–53), altered placental vascular pathology (54), cesarean section rates (55), glucose intolerance (56), adverse birth outcomes due to race and ethnicity (57), brain dysfunction (50) and respiratory dysfunction (38). Since 1980, studies have shown that maternal vitamin D deficiency is a variety of adverse health outcomes, which include abnormal fetal growth patterns (with the likelihood of alteration in growth associated with extreme deficiency) (58), adverse birth outcomes (such as preterm birth) (59, 60), reproductive failure (61–65), and have further strengthened vitamin D's role as a contributing factor in the manifestation and progression of disease leading to preeclampsia (66, 67). A recent meta-analysis of observational studies has found a positive relationship between maternal vitamin D deficiency and the risk of preterm birth (68).

Changing public awareness about vitamin D involves several steps, the first of which has been the publication of observational studies of vitamin D deficiency, alerting scientists and others that vitamin D deficiency has subtle but potentially profound effects. The next step was the conduct of randomized controlled trials, but unlike a pharmaceutical trial where everyone starts off at a baseline concentration of the said drug of 0; in nutrient studies, in this case vitamin D, each study participant has a measurable amount of circulating 25(OH)D, which certainly confounds any such trial. As Dr. Robert Heaney pointed out in his essay on clinical nutrient studies, the importance of a biomarker of a drug, in this case “vitamin” or preprohormone, is underscored (69). Analyses of the effect of vitamin D therapies should use 25(OH)D concentration, and not treatment group, as the better indicator of true effect.

### Randomized clinical trials

As the gold standards, evidence-based medicine (EBM) and randomized clinical trials are long considered essential in advancing health interventions and practices. This approach has and specific methods has been applied to the evaluation of nutrient effects. As mentioned above, in advancing the cause of nutrient studies, Heaney (69) pointed out that EBM, while appropriate in the evaluation of drugs, is lacking when applied to the study of nutrients. For example, in a clinical trial designed to study a given drug's efficacy, the placebo group would not have been exposed to that drug and would receive none of the drug in question. This is not the case for nutrient studies, including vitamin D studies. To perform a true RCT for vitamin D, the study design would ensure that all subjects were vitamin D-deficient at the study onset, and for the duration of the study, all subjects would have to remain indoors to avoid any sun exposure. In fact, places such as the Middle East where women for cultural and religious reasons have limited sunlight exposure and are not likely to receive vitamin D supplementation, profound vitamin D deficiency exists, and when those women are randomized to vitamin D treatment, there are profound effects noted (70–72). In other regions of the world where vitamin D supplementation is given to pregnant women, a true placebo would be considered unethical, and thus, the dilemma surrounding nutrient study design and interpretation of the data exists.

The five rules of rigor for nutrient studies suggested by Heaney (69) include the following: (1) basal nutrient status must be measured, used as an inclusion criterion for entry into the study, and recorded in the report of the trial; (2) the intervention must be large enough to change nutrient status and must be quantified by suitable analysis; (3) the change in nutrient status produced in those enrolled in the report of the trial must be measured and reported; (4) the hypothesis to be tested must be that a change in nutrient status produces the sought-after-effect; and (5) the status of other nutrients must be optimized to guarantee that the nutrient being studied is the only nutrition-related, limiting factor in the response. We also have added an additional imperative to this list: the nutrient being investigated has to follow an appropriate dosing schedule matching the physiologic system being investigated, as for example with vitamin D, there is a substantial physiological difference between daily, weekly and monthly dosing (73). Most vitamin D clinical trials conducted thus far would fail based on at least two of these criteria. We thus are forced to look at observational and RCT data that have nutrient study “flaws,” but which offer important insights into vitamin D's role in health.

The first studies involving vitamin D supplementation in pregnant women were performed in the early 1980's mainly in Europe (58). These early studies were plagued by small sample sizes, not having meaningful endpoints, or effective treatment/dosing concentrations (58) that could not effectively evaluate the role of vitamin D in the development of certain disease states such as preeclampsia (13, 51–53), asthma (38, 39, 74–76), preterm birth (59, 60, 68, 77), and autoimmune dysfunction (78–82). As a result, the variability in findings led to confusion and limited relevance to the general population, which resulted in stagnation of the field for at least two decades.

As one of the first steps in discerning what is a reasonable, healthy concentration of 25(OH)D, our group in 2001 designed a large RCT to assess the vitamin D requirements during pregnancy. This study represented a radical departure from prior studies in that we proposed a randomized clinical trial of supplementing pregnant women <16 weeks of gestation with up to 4,000 IU/d vitamin D_3_ until delivery. The higher dose treatment group was two times the Upper Limit (UL) set forth by the IOM in 1997 (83). Our main goal of the study was to determine the daily dose of vitamin D required to raise circulating maternal 25(OH)D concentrations to at least 80 nmol/L (32 ng/mL) by the third trimester, which, based on mathematical calculations from previous studies (6, 84), was the amount necessary to prevent secondary hyperparathyroidism (85).

Along with our study, several other RCTs have been published (11, 86–95), with the main finding that a daily dose of vitamin D of 4,000 IU/safely elevated circulating 25(OH)D concentrations that, regardless of race, fully and safely normalized vitamin D metabolism and calcium homeostasis in the pregnant women. Using repeated measures, the concentration of 25(OH)D that fully normalized 1,25(OH)_2_D in our study cohort was determined on each subject and plotted to determine the point at which first order kinetics went to zero order (11), which was 100 nmol/L (40 ng/mL), the beginning point of the plateau at which the production of 1,25(OH)_2_D became substrate independent (11). Attention to safety in our study as well as other studies showed that serum calcium and urinary calcium/creatinine ratios did not differ between the treatment groups, and thus, 4,000 IU/day vitamin D was deemed to be safe (11, 86–95).

With the emergence from observational vitamin D studies of the favorable efforts of vitamin D on pregnancy outcomes beyond calcium homeostasis, we analyzed health outcomes measures in our pregnancy cohorts. Improved vitamin D status was associated with decreased complications of pregnancy and lower rates of cesarean section (77, 87). Merewood et al previously had shown a similar association between vitamin D and mode of delivery in a cohort of women living in Boston (55). Further, RCT data and analysis by our group and others in various regions of the world have clearly demonstrated that higher doses of vitamin D during pregnancy improve birth outcome data (59, 72, 77, 89, 96).

The list of studies that links vitamin D deficiency to complications of pregnancy continues to grow: vitamin D status has been associated with gestational diabetes (56, 89, 90, 92), aeroallergen sensitization (91), and markers of regulatory immunity (93). Perhaps one of the most far-reaching of these studies was performed by Sablok et al. (89). In their study, vitamin D deficient pregnant women living in Delhi, India, with circulating 25(OH)D concentrations of <25 nmol/L (10 ng/mL), were randomized to receive substantial amounts of vitamin D starting at 20 weeks of gestation or placebo. It is noteworthy that the placebo group remained profoundly vitamin D deficient throughout pregnancy. In the women randomized to vitamin D treatment, there was 100% adherence to protocol, which resulted in a substantial decline in pregnancy complications. In other areas of the world where all women receive at least 400 IU vitamin D/day, there is less deficiency and the differences in clinical outcomes less dramatic. Thus, the effects of vitamin D deficiency appear to be magnified in the cases of severest deficiency, which is the end result of no vitamin D supplementation (placebo).

## Vitamin D-induced genomic alterations during pregnancy

In our original pregnancy study and a subsequent study, from the perspective of an intention-to-treat design, the results are less dramatic (11, 97) than when adherence is taken into account. If instead, circulating 25(OH)D concentrations as the biomarker of vitamin D status is used, the true effect of vitamin D supplementation on preterm birth becomes apparent (59). The same associations from the VDAART trial also hold true for the prevention of preeclampsia (17). Through vitamin D's effect on gene regulation, vitamin D supplementation during pregnancy appears to alter genes related to systemic inflammation and immune responses. The aberration of these genes and processes suggests that there is a specific immune cascade of events associated with vitamin D deficiency that occurs early-on in pregnancy in women destined to develop preeclampsia (16, 65), and likewise, in other comorbidities states of pregnancy such as gestational diabetes (90, 92, 98) and infection (99, 100).

In their recent paper, Al-Garawi et al. (101), in their *post-hoc* analysis of the VDAART RCT study, provide strong evidence of vitamin D's effect on genomic changes during pregnancy, which is one of the first reports of its kind. As part of the parent RCT of vitamin D supplementation in pregnancy at 10–18 weeks of gestation where women were randomized to 400 and 4,400 IU vitamin D_3_/day to achieve decreased pediatric asthma risk (86), a subset of blood samples also were collected for RNA analyses for gene expression in the first and third trimesters, for comparison. Using significance of analysis of microarrays (SAM) and clustered weighted gene co-expression network analysis (WGCNA) to identify major biological transcriptional profiles between first and third trimesters of pregnancy, this team of investigators identified 5,839 significantly differentially expressed genes. Transcripts from these genes clustered into 14 co-expression modules, of which two showed significant correlation with maternal 25(OH)D concentrations. Two modules identified genes enriched in immune defense pathways and extracellular matrix reorganization as well as genes enriched in Notch signaling and transcription factor networks. These important findings suggest that maternal gene expression changes during pregnancy are affected by maternal vitamin D status, which in turn, is a direct reflection of maternal vitamin D supplementation.

The extrapolation of US- and European-based studies to other regions of the world is based on the effects of vitamin D that extend beyond calcium and bone metabolism, and on the findings that vitamin D deficiency impacts immunological function and pregnancy outcomes. Whether a woman attains optimal vitamin D status during pregnancy through sun-exposure or vitamin D supplementation is less of an issue than if she is able to attain optimal status by either method such that her circulating 25(OH)D concentrations are at least 100 nmol/L (40 ng/mL). Women who have profound vitamin D deficiency typically respond to 4,000 IU/day with an exuberant response in the first month since they have upregulated their vitamin D enzymes but by 2 months of supplementation, we have found that their 25(OH)D concentrations will have plateaued and remain so throughout pregnancy. Even these women with initial severe deficiency do not develop hypercalcemia or hypercalciuria in response to treatment (11, 77, 97). If a woman has a higher BMI, she may require additional vitamin D to achieve this goal concentration; or if she is non-compliant with treatment, her vitamin D status will not improve. It is important to follow women with serial 25(OH)D measurements if such risk factors are identified.

There are so many unanswered questions that are the focus of ongoing studies. For example, what effect does maternal 25(OH)D concentration have on fetal development? Are these effects direct or through downstream processes? What about direct effects of maternal gene expression on the fetus? Studies presented at recent vitamin D conferences suggest that there are indeed direct genomic alterations in response to maternal vitamin D status that can alter the health of the mother and birth outcomes (102).

### Postnatal asthma prevention

Observational studies by Brehm et al suggested that vitamin D supplementation during pregnancy could reduce childhood asthma rates (102). This led to a double-blind multicenter RCT conducted in the US where pregnant women were randomized to 400 or 4,400 IU/d vitamin D_3_ across the three major racial/ethnic groups in the US from 10 to 18 weeks of gestation until delivery (the VDAART study) (86). As the authors describe, the primary endpoint—prevention of asthma/wheeze in the infant/child at 1-, 2-, 3-, and 6-years post-birth through vitamin D supplementation in the mother during pregnancy—involved nearly 900 high-risk subjects (86). The results of this study are quite clear: on the basis of intention-to-treat, where there was much non-adherence to protocol there is a strong trend of effect (*p* = 0.051); however, on the basis of maternal circulating 25(OH)D concentrations achieved, vitamin D supplementation during pregnancy will decrease asthma or recurrent wheezing rates in children. This positive finding, however, is dependent upon a circulating 25(OH)D concentration of at least 100 nmol/L (40 ng/mL) at conception (39, 74, 86).

In their RCT study performed in Denmark, Wolsk et al also examined the effect of maternal vitamin D supplementation during pregnancy on later asthma risk in the offspring (75). The Denmark team found results similar to the VDAART study. Combining the VDAART data with the Denmark data (74), the two study teams found that vitamin D_3_ given to a pregnant woman reduced the risk of later asthma/wheeze in her child (39, 74).

The wealth of data in the VDAART study led to additional *post-hoc* analyses (39, 74). Those women entering pregnancy with circulating 25(OH)D concentrations ≥75 nmol/L (30 ng/mL) who were prescribed 4,000 IU/d vitamin D_3_ starting at approximately 10–18 weeks' gestation achieved the maximum protection against asthma development in their infants following birth (39, 74). Further, these data suggest that vitamin D is strongly associated with very early *in utero* lung development in the fetus that cannot be reduced by starting vitamin D supplementation at the end of the first trimester. As discussed earlier, vitamin D-related genes in early lung development are associated with asthma pathogenesis (35).

Are these data from the various studies in the US, Europe, and Iran applicable to other regions of the world? Is there something unique about women in these countries or can the results be extended to women in all regions of the world if they have limited sunlight exposure either through where they live or how they live? Based on vitamin D metabolism and the pharmacokinetics of vitamin D, we believe that the recommendations of 4,000 IU/day during pregnancy should be universal, with the caveat that women with higher BMIs may require higher dosing to attain the optimal 25(OH)D concentration of at least 100 nmol/L (40 ng/mL).

### Preeclampsia prevention

In their *post-hoc* analysis derived from the VDAART RCT, Mirzakhani et al. (17) reported that a key factor in preventing preeclampsia was vitamin D status early in pregnancy: first trimester circulating 25(OH)D concentration of a least 100 nmol/L (40 ng/mL) was associated with a reduction in the risk of developing preeclampsia. Predicted by both observational studies as well as experimental animal models, the association of vitamin D deficiency and its ability to alter placental development and embryo implantation is further supported by the findings of Mirzakhani et al. (15, 17, 103, 104). As mentioned earlier, this effect is likely vitamin D-mediated during a very early point in pregnancy. Beyond this critical period, rescue by further vitamin D supplementation with respect to placentation and the manifestation of preeclampsia, has diminished impact (17). Additional support for vitamin D's role in the development of preeclampsia comes from a recent RCT study where the administration of vitamin D (50,000 IU/week or ~7,142 IU/day) during the prenatal period reduced the pre-eclamptic rate by half (95).

It is not surprising based on vitamin D's effect on gene regulation that maternal vitamin D supplementation also would be involved in epigenetic regulation. Pathways affected by such regulation include antigen processing and presentation, inflammation, regulation of cell death, cell proliferation, transmission of nerve impulse, neurogenesis, neuron differentiation, sensory organ development (105) and vitamin D metabolism (106). Vitamin D's epigenetic effects on genes involved in metabolism and immune function have been demonstrated in experimental animal models (107, 108) and preliminary findings have been reported in humans (105). The effect of vitamin D status on total genomic changes cannot be ignored and represent one of the most exciting branches of research to be explored.

### Neurodevelopment and autoimmune consequences

There is emerging data regarding the impact of vitamin D deficiency during pregnancy on neurologic disease and altered development (30, 49, 50). Experimental animal data suggests that there are significant adverse neurological consequences to the offspring if vitamin D is restricted during pregnancy (109–112). A review by Patrick and Ames summarizes cumulative data that support the premise of adverse effects of intrauterine vitamin D deficiency. Vitamin D likely acts through the control of serotonin synthesis in the developing brain: if vitamin D access is restricted, can lead to the later development of autism, attention deficit disorder, bipolar disorder, schizophrenia and impulse behavior (113). A recent study again strongly links vitamin D concentrations during pregnancy with the development of autism spectrum disorder (114).

## Current recommendation for vitamin D supplementation during pregnancy

Based on our data from our NICHD-sponsored vitamin D supplementation pregnancy trial (11) as well as substantial observational and interventional data from our later studies (16, 60, 97) and that of other investigators around the world (17, 39, 72, 74, 86, 94, 101), we suggest that women considering becoming pregnant (59), and if pregnant, then during the earliest time in pregnancy, maintain a circulating 25(OH)D concentration of a least 100 nmol/L (40 ng/mL). Achieving this goal will reduce the risk of vitamin D-related pregnancy complications, including preeclampsia (17, 115) and preterm birth (60, 97), and later asthma risk in the offspring (17). To achieve this goal, intakes of a least 4,000 IU/d vitamin D_3_ will be required because of variable individual abilities to convert vitamin D to 25(OH)D (6). These supplements have proven to be safe in thousands of patients over the past 15 years. Further, this supplementation dose is well within the safe intake level (upper limit, UL) as defined by The Endocrine Society (8). Such supplementation becomes an alternative to direct sunlight exposure, and agrees with data derived from populations living in sun-rich environments (116), whose circulating 25(OH)D concentrations during pregnancy simply from sun exposure are quite similar to those achieved through daily vitamin D supplementation of 4,000 IU (116, 117).

Finally, addressing the original question of this review—does vitamin D qualify as a substance that supports and upholds the Barker Hypothesis? The clear answer is yes! Through its effect on genetic processes, vitamin D deficiency during pregnancy affects both mother and fetus, and, at least in the case of asthma, phenotypic expression in the infant/child.

In summary, the observational and randomized clinical trials present a clear message: that 4,000 IU/d vitamin D_3_ supplementation is beneficial to both mother and her developing fetus through optimization of vitamin D metabolism that goes beyond classical calcium and bone homeostasis. While further work is needed to determine what the optimal dose of vitamin D supplementation during pregnancy is based on various genotype differences for the vitamin D binding protein and the vitamin D receptor, body mass index, status at the time of conception, and other factors such as sunlight exposure and latitude, based on our work and that of others, we believe that all individuals, including pregnant women, should achieve a target circulating 25(OH)D concentration of 100 nmol/L (40 ng/mL) as early as possible. Because of individual differences in what is required to attain this target concentration of 25(OH)D, we believe all women should consume at least 4,000 IU/d vitamin D_3_ prior to conception and throughout pregnancy.

## Author contributions

All authors listed have made a substantial, direct and intellectual contribution to the work, and approved it for publication.

### Conflict of interest statement

The authors declare that the research was conducted in the absence of any commercial or financial relationships that could be construed as a potential conflict of interest. The handling Editor and reviewer PP declared their involvement as co-editors in the Research Topic, and confirm the absence of any other collaboration.
